# Personal

**Published:** 1892-03

**Authors:** 


### Personal.

---

Dr. A. F. O'Hara has been appointed keeper of the Quarantine Hospital under the new health régime. Dr. Wende is exercising rare skill and judgment in the selection of his appointees, and this last mentioned one is to be commended on all hands as an improvement over the former system, whereby a non-professional man was kept in charge of that department of the public health service.

---

Dr. Richard A. Heath, of Wadsworth street, is the first physician in Buffalo to obtain the State license to practise medicine and surgery under the recent law creating a State Medical Examining and Licensing Board. Dr. Heath also enjoys the further distinction of being the only physician in Buffalo to possess a State license up to the present date. Dr. Heath is not an exempt.

---

Dr. Wm. C. Krauss, Professor of Pathology, and Dr. John A. Miller, Professor of Chemistry, in the Medical Department of the Niagara University, have been elected Fellows of the Royal Microscopical Society of London.

---

Dr. Walter Coles was lately elected President of the St. Louis Medical Society for the ensuing year. The Society is to be congratulated upon this admirable selection.

Dr. Wm. B. Southard was recently chosen President of the Kalamazoo (Michigan) Academy of Medicine. The annual meeting of the Academy was held February 4th, at which time Dr. Heneage Gibbes, of Ann Arbor, read a paper on Formation of Cavities in the Lungs. It was an ample discussion of this important and interesting subject.

---

Dr. William H. Heath has been appointed inspector of food and drugs by the Health Commissioner, Dr. Ernest Wende. Whatever can be done in the official inspection to give the citizens of Buffalo pure food and drugs, will be done under the present administration of the Health Department, as instanced by this excellent appointment.

---

Dr. Byron Stanton was elected President of the Cincinnati Obstetrical Society for the ensuing year, at the last annual meeting of that efficient medical organization. This is a fitting honor for the Society to bestow upon one of its most faithful and capable members.

